# Strengthening health disaster risk management in Africa: multi-sectoral and people-centred approaches are required in the post-Hyogo Framework of Action era

**DOI:** 10.1186/s12889-016-3390-5

**Published:** 2016-08-02

**Authors:** Olushayo Olu, Abdulmumini Usman, Lucien Manga, Stella Anyangwe, Kalula Kalambay, Ngoy Nsenga, Solomon Woldetsadik, Craig Hampton, Francois Nguessan, Angela Benson

**Affiliations:** 1 WHO Country Office, PO 1324 Kigali, Rwanda; 2 WHO Country Office, Juba South, Sudan; 3 WHO Regional Office for Africa (AFRO), Brazzaville, Congo; 4 School of Health Systems and Public Health, University of Pretoria, Pretoria, South Africa; 5 International Public Health Disaster Risk Management Consultant, Gatineau, Canada; 6 WHO Intercountry Support Team for Western Africa, Ouagadougou, Burkina Faso; 7 WHO Country Office, Kampala, Uganda; 8 International Public Health and Disaster Risk Management Consultant, Monrovia, Liberia

**Keywords:** Health, Disaster risk management, Africa, Health system, Hyogo Framework for Action, Disaster risk reduction, Sendai

## Abstract

**Background:**

In November 2012, the 62nd session of the Regional Committee for Africa adopted a comprehensive 10-year regional strategy for health disaster risk management (DRM). This was intended to operationalize the World Health Organization’s core commitments to health DRM and the Hyogo Framework for Action 2005–2015 in the health sectors of the 47 African member states. This study reported the formative evaluation of the strategy, including evaluation of the progress in achieving nine targets (expected to be achieved incrementally by 2014, 2017, and 2022). We proposed recommendations for accelerating the strategy’s implementation within the Sendai Framework for Disaster Risk Reduction.

**Methods:**

This study used a mixed methods design. A cross-sectional quantitative survey was conducted along with a review of available reports and information on the implementation of the strategy. A review meeting to discuss and finalize the study findings was also conducted.

**Results:**

In total, 58 % of the countries assessed had established DRM coordination units within their Ministry of Health (MOH). Most had dedicated MOH DRM staff (88 %) and national-level DRM committees (71 %). Only 14 (58 %) of the countries had health DRM subcommittees using a multi-sectoral disaster risk reduction platform. Less than 40 % had conducted surveys such as disaster risk analysis, hospital safety index, and mapping of health resources availability. Key challenges in implementing the strategy were inadequate political will and commitment resulting in poor funding for health DRM, weak health systems, and a dearth of scientific evidence on mainstreaming DRM and disaster risk reduction in longer-term health system development programs.

**Conclusions:**

Implementation of the strategy was behind anticipated targets despite some positive outcomes, such as an increase in the number of countries with health DRM incorporated in their national health legislation, MOH DRM units, and functional health sub-committees within national DRM committees. Health system-based, multi-sectoral, and people-centred approaches are proposed to accelerate implementation of the strategy in the post-Hyogo Framework of Action era.

## Background

The United Nations defines disaster as *“a serious disruption of the functioning of a community or a society involving widespread human, material, economic or environmental losses and impacts, which exceeds the ability of the affected community or society to cope using its own resources”*[1]. In the last three decades, Africa has borne a significant disaster burden, largely due to the effects of climate change, environmental degradation, rapid urbanization, increasing poverty, and increased disputes over land, resources, ethnic identity, and political and religious ideology [2, 3]. Of the newly displaced 11 million people globally in 2014, an estimated 60 % were from five countries, three of which were African countries (South Sudan, Democratic Republic of Congo, and Nigeria) [3]. In addition, 77 % of all internally displaced persons worldwide live in 10 countries, five of which are African countries. Of the 30 biggest complex emergencies and epidemics that occurred globally between 1995 and 2004, Africa accounted for 17 emergencies and 25 epidemics [4]. The 2014/15 outbreak of Ebola virus disease (EVD) in West Africa, one of the most recent disasters on the continent, resulted in the infection of 28,610 people and 11,308 (39.5 %) deaths in the three principally affected countries namely Guinea, Liberia and Sierra Leone, making it unprecedented and the largest outbreak in the history of the disease [5]. The EVD outbreak resulted in the death of hundreds of health workers, disrupting health services delivery and reducing gross domestic product by 3–13 %, which negatively impacted on the economy of the countries principally affected (Guinea, Liberia, and Sierra Leone) [6, 7]. The impact of disasters on public health and the health systems of affected populations include destruction of health facilities, looting of essential medicines and medical equipment, displacement and death of healthcare workers (resulting in further disruption of health services delivery) [8, 9], communicable diseases outbreaks [10], mental health and psychosocial problems [11], and increased malnutrition.

Until now, the management of disasters has been limited to preparedness and response, with little understanding of the need for risk reduction and post-disaster recovery. However, the 2005 introduction of the Hyogo Framework for Action (HFA) 2005–2015 resulted in a global paradigm shift from limited emergency actions to more comprehensive approaches to disaster management [12]. The Sendai Framework for Disaster Risk Reduction (SFDRR) 2015–2030, which succeeded the HFA 2005–2015, reinforced the need for a broad approach to disaster risk management (DRM). The SFDRR emphasized the need to strengthen health disaster risk reduction (DRR) and stipulated the strengthening of national health systems as a means of achieving DRR [13].

Drawing from experiences in responding to disasters and consistent with HFA principles, the World Health Organization (WHO) recognized the importance of an all-hazard and whole-of-health approach for health sector disaster management. The WHO articulated its core commitments to health DRM in the World Health Assembly Resolutions 64.10 [14] and 65.20 [15]. In November 2012, the 62nd session of the Regional Committee for Africa adopted a comprehensive 10-year regional strategy for health DRM to operationalize the WHO’s core commitments to health DRM and the HFA 2005–2015 within the health sectors of its 47 African region^1^ member states (MS) [16]. The strategy aimed to contribute to the reduction of disaster risks using a multidisciplinary approach to build health system and community resilience. More specifically, it intended to ensure availability of relevant policies, strategies, and capacity to guide health sector DRM interventions, reduce the occurrence and number of emergencies that progress to disasters, and improve risk management capacities as well as preparedness, responsiveness and recovery [16].

**Fig. 1:**
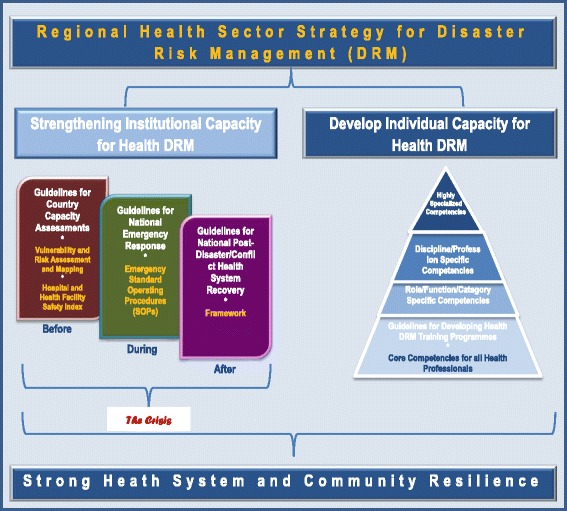
Diagrammatic illustration of the African regional health sector strategy for disaster risk management

The strategy has six components:

1. Establishment of an institutional framework for health DRM;
2. Establishment of adequate health sector capacity for risk management;
3. Establishment of a relevant framework and capacity for assessment and mapping of health risks and vulnerabilities;
4. Implementation of resilience-building interventions in health facilities and at a community level;
5. Strengthening systems for adequate preparation, and timely and adequate response to and recovery from disasters; and,
6. Generation and dissemination of relevant information on hazards and their impact on public health (Fig. 1).

The strategy sets out nine main targets that are expected to be achieved incrementally by 2014, 2017, and 2022, with evaluations at each time point (Table 1).

**Table 1:** Nine regional health sector strategy targets for disaster risk management

Targets to be achieved by all member states (MS) by the end of 2014: 1. Establishment of a functional and equipped unit within the Ministry of Health to coordinate implementation of health sector disaster risk management (DRM) interventions; 2. Establishment of functional health sector subcommittees within national multi-sectoral DRM coordination committees; 3. Incorporation of DRM into national health legislation, national health policies, and health sector strategic plans; 4. Health disaster risk analysis and mapping using a multi-sectoral approach. Targets to be achieved by all MS by the end of 2017: 5. A health preparedness planning and management process that includes planning, stockpiling essential supplies, resource allocation, emergency simulations, and regular updating of all risks in the country; 6. Incorporation of emergency and disaster early warning, preparedness, response, and recovery indicators in national disease surveillance and health information management systems; 7. Health facility safety and community resilience building based on disaster risk analysis and mapping; 8. Establishment of emergency and disaster response and recovery operations. Targets to be achieved by all MS by the end of 2022: 9. All MS will have fully implemented the interventions set out in the regional strategy.	

This study reports the findings of the formative evaluation of the implementation of the strategy. The objectives were to: 1) evaluate the progress in achievement of the nine targets; 2) document the lessons learnt so far; and, 3) propose recommendations for accelerating implementation of the strategy to operationalize the SFDRR within the health sectors of African countries.

## Methods

We evaluated the implementation of the African regional health sector strategy for DRM from its inception in 2012 to 2015. Mixed data collection methods were used to obtain quantitative and qualitative information. A cross-sectional quantitative survey was conducted to assess progress toward achievement of the nine regional strategy targets in each of the African MS. Available reports and information on the implementation of the strategy were assessed, and a review meeting was held to discuss and finalize the study findings.

For the quantitative cross-sectional survey, a questionnaire was designed, pretested, and administered to DRM focal point staff within the Ministries of Health (MOH) in all 47 MS in the first half of 2015. The questionnaire included 13 yes/no questions to assess the implementation of the nine targets, and collected information on strengths, weaknesses, opportunities, and threats to the implementation of the strategy. The questions concerned the availability of DRM units within MOHs, existence of national coordination mechanisms, DRM legislation, risk assessments, early warning systems, and preparedness and response planning systems. Staff members from each of the 47 MOHs were requested to complete and send the filled questionnaire to the WHO Regional Office for Africa (WHO/AFRO), with a brief narrative report. To ensure a good response rate, two messages were sent to the MOHs to remind them to complete and send the questionnaires. Completed questionnaires were validated by WHO outbreak and disaster management (ODM) officers at the country offices. Survey data were entered into a Microsoft Excel database, cleaned, and analyzed by calculating the frequencies of the responses to all questions.

The review of available reports and information on the implementation of the strategy drew on information obtained from available literature on DRM and DRR in general, and as they related to the health sector in particular. A list of documents, declarations, resolutions, guidelines, and reports associated with the implementation of the HFA, SFDRR, and the regional strategy was developed via online searches (using PubMed and Google search engines), compiled, and reviewed. The terms used for the PubMed search was “health and disaster risk reduction and Sendai” while “health and disaster risk management and HFA” and “Sendai and health and disaster risk management” were used for Google search.

The documents included HFA and SFDRR strategic documents and reports, African regional health sector strategy for DRM documents, reports of meetings and implementation of key interventions in the strategy, and existing literature on health DRM. All of the authors had participated in the implementation of the strategy in various capacities and at various times, and observed and gathered information on the strengths, challenges, opportunities, and threats relating to the implementation of the strategy.

A review workshop was convened from 21 to 25 September, 2015 in Yaoundé, Cameroon. The meeting was attended by 10 WHO ODM staff from the WHO country offices, WHO/AFRO and headquarter. This workshop aimed to:

1. Analyze data from the questionnaires and reports;
2. Discuss and agree on the level of achievement of the regional strategy targets at the regional level using the outcomes of the quantitative survey;
3. Discuss and agree on the key strengths, weaknesses, challenges, and opportunities relating to implementation of the DRM strategy in the region; and,
4. Propose recommendations to accelerate implementation of the DRM strategy in the context of the SFDRR.

## Results

Of the 47 African MS, 25 completed and returned questionnaires, giving a response rate of 53 %. Many of the assessed countries (58 %) had staffed and funded DRM coordination units within their MOH, and 24 % (6 countries) had coordination units but no dedicated resources such as funds. Most MOHs had dedicated DRM focal staff (88 %) and national-level DRM committees (71 %) (Table 2). Fourteen (58 %) of the responding countries had health DRM subcommittees on a multi-sectoral DRR platform. Only a few countries had incorporated components of DRM in their legislation and national health policies, although many (54 %) had included DRM in their national health strategic plans. Risk analysis and mapping were weak in most of the assessed countries; less than 40 % had conducted disaster risk analysis, hospital safety index, and Health Resources Availability Mapping System (HeRAMS) surveys. Only one country (Tanzania) had conducted a full health sector Vulnerability and Risk Analysis and Mapping (VRAM) assessment. National health emergency preparedness plans were available in 58 % of countries, contingency supplies such as essential medicines, medical supplies, equipment were available in 13 countries, and 46 % of countries had conducted an emergency simulation exercise for their plan. National all-hazard policies and plans were available in nine (38 %) of the countries assessed. As of January 2016, nine (19 %) of the 47 countries in the African region had conducted DRM country capacity assessments (CCA), and had developed and were implementing health DRM strengthening roadmaps (Sierra Leone, Tanzania, Uganda, Ethiopia, the Democratic Republic of Congo, Kenya, Seychelles, Ethiopia and Gambia).

**Table 2:** Summary of regional progress in key areas of the health sector strategy for disaster risk management in the African region as at May 2015

Health DRM Strategy component	Variable	N (%)
Coordination	DRM coordination units within the MOH	14 (58)
	DRM focal staff within the MOH	21 (88)
	DRM committees established	17 (71)
	Health DRM subcommittees established	14 (58)
Inclusion of DRM in national policies	DRM in legislation	10 (42)
	DRM in health policies	4 (17)
	DRM health strategic plans available	13 (54)
Risk analysis and mapping	Disaster risk analysis conducted	9 (38)
	Hospital safety index conducted	3 (13)
	HeRAMS conducted	6 (25)
Preparedness planning	National preparedness plan available	14 (58)
	Contingency supplies available	13 (54)
	Simulation exercises conducted	11 (46)
All-hazard strategies and policies	National all hazard policies and plans available	9 (38)

*DRM* disaster risk management, *HeRAMS* Health Resources Availability Mapping System, *MOH* Ministry of Health

The review of available reports and information found that all of the tools and guidelines required to support MS in effectively implementing the strategy had been developed. In total, five guidelines aimed at strengthening health interventions during the pre-, intra-, and post-disaster phases had been developed: health DRM CCA, conducting VRAM in the health sector, developing health sector emergency standard operating procedures, post-disaster/conflict health system recovery, and developing health DRM health training for African health workers.

The key findings of the review of available information and reports were categorized as strengths, weaknesses, opportunities, and threats associated with the implementation of the strategy (Table 3).

**Table 3:** Summary of strengths, weaknesses, opportunities, and threats affecting health disaster risk management and implementation of the regional strategy

Strengths	Weaknesses
1. Some countries had strong support from a WHO country office, with MOH prioritization of DRM and national disaster management authorities 2. DRM incorporated into national coordination forums and included in national preparedness plans, health development plans, and the United Nations development assistance framework in some countries 3. Presence of health DRM focal points (staff) in some WHO country offices and MOHs; 4. Availability of the tools required for implementation of the health DRM strategy 5. Increased awareness about health DRM among MS	1. Little importance attached to health DRM: lack of political commitment and resource allocation for DRM 2. Multiple plans for specific hazards; most health plans focused on specific disease/epidemics and were not integrated into all-hazard disaster strategies 3. Health DRM planning not guided by outcomes of health risk assessments 4. Lack of a multi-sectoral approach to implementation of health DRM 5. Weak health systems and limited human resources capacity in most MS 6. Disconnect between elements of DRM (e.g., risk awareness, preparedness, surveillance, and response) 7. Limited practical exercises in managing emergency responses 8. Inadequate engagement and mobilization of communities for health DRM implementation
Opportunities	Threats
1. Emphasis on health in the SFDRR 2. Availability of climate change adaptation resources, initiatives, and plans that can be leveraged for health DRM 3. Availability of other programs/projects such as the International Health Regulations and the Global Health Security Agenda that support implementation of the health DRM strategy 4. Ongoing emergency reforms in the WHO that consider health DRM	1. Increasing risks and emergencies in the African region and globally that drain health DRM resources 2. Non-compliance with the principles of the health DRM strategy, meaning implementation of health DRM policies and frameworks may not translate into improved capacities and actions 3. Unstable political environment; violence and insecurity in most of the disaster prone countries in the region 4. Inadequate supervision and monitoring of health DRM activities 5. Insufficient funding, resources, and dedicated DRM staff 6. Unavailability of scientific evidence on the nexus between health systems and DRM

*DRM* disaster risk management, *MOH* Ministry of Health, *MS* member states, *WHO* World Health Organization

## Discussion

To our knowledge, the African strategy for health DRM is the first sector-wide regional strategy in the African Region. It represents a significant shift from limited emergency preparedness and response action to a more comprehensive disaster risk management approach that includes risk reduction, preparedness, response, relief, and recovery within the health sector. Health emergency and national DRR platform managers from 31 of the 47 African MS were briefed and sensitized about the strategy in early 2013. After this briefing, these countries developed operational plans for implementing the strategy.

An African Public Health Emergency Fund (APHEF), managed by the WHO on behalf of African MS, was established in 2012 [17]. APHEF supports investigation and response activities for disease outbreaks and public health emergencies in the African Region. Funding support follows formal declaration of the outbreak or public health emergency by the affected MS, the appointment of a humanitarian coordinator, or a humanitarian appeal launched by the affected MS. The fund is financed through an annual voluntary contribution from WHO/AFRO MS of US$ 50 million. Funding is limited to a maximum of US$2 M per emergency per country. Since its inception, 13 of the 47 MS have contributed a total of US$ 3.6 million, with a contribution of US$ 196, 380, 562 pending; this amount represents only 1.8 % of the US$ 200 million in contributions expected for that period [18].

Our study found modest progress in the implementation of the regional strategy. Marginal improvements attributable to its implementation were observed in countries with health DRM incorporated into their national health legislation, MOH DRM units, and health sub-committees within their national DRM committees. Implementation of the strategy also resulted in increased awareness about health and DRM among the MS in the region. The percentage of emergency health simulations conducted in the region increased from 19 to 46 % in the 3-year period during which the strategy has been implemented, and hospital safety index assessment was conducted in the region for the first time.

However, these modest achievements fall short of the targets MS were expected to achieve by 2014: 1) establishment of DRM coordination units in all MOHs; 2) establishment of functional health sector sub-committees in all national DRM coordination platforms; and, 3) incorporation of DRM in all national health legislation, policies, and strategic plans. Our study identified some challenges that were responsible for the slow rate of strategy implementation.

First, inadequate political will and commitment of MS was a critical factor that continued to limit the implementation of the strategy. This resulted in inadequate resources for DRM in the MS and low contributions to the APHEF. The contribution of only 3 % of the annual target of APHEF contribution is grossly inadequate to fund the increasing number of public health emergencies the region experiences. While the APHEF has been instrumental in supporting many MS to respond to acute emergencies, due to its mandate it has been unable to support other critical components of DRM such as DRR and preparedness. Second, the inherently weak health systems in the region present major challenges in effectively implementing the strategy. For example, the poor human resources for health, weak health information and disease surveillance systems, fragmented supply chains for essential medicines and medical supplies, weak health governance structures, and inadequate health financing prevalent in the West African countries affected by the 2014/15 EVD outbreak severely compromised their capacity to detect and adequately respond to the outbreak [19]. Similar experiences were also observed in other crises such as the Horn of Africa and Sahel drought and the armed conflicts in South Sudan, the Central Africa Republic, and Mali.

Third, poor community engagement and participation in health DRM and DRR were a critical challenge, as evidenced by poor community participation in the response to emergencies such as the West Africa EVD outbreak [20]. This was perhaps one of the most important factors responsible for the scale and long duration of the outbreak [21]. Fourth, there was a lack of relevant policies enabling technical environments and lack of an all-hazard and whole-of-health approach to disaster planning, which often resulted in fragmentation of health DRM activities. Fifth, the dearth of scientific evidence on mainstreaming DRM and DRR in longer-term health system strengthening and development programs, and lack of health disaster risk analysis information to guide effective health emergency planning were also identified as challenges.

Our study identified several pertinent lessons and opportunities that may facilitate implementation of the regional strategy and operationalization of the SFDRR in the health sectors of African countries. The CCAs revealed critical gaps which should be addressed to facilitate the smooth implementation of the strategy. These included weak capacity for health disaster vulnerability and risk mapping, limited community-level health DRM initiatives, lack of hospital safety and resilience-building programs, and insufficient human capacity for health DRM. The CCAs also highlighted the existence of several global, regional, and national DRM initiatives containing elements of health DRM. This suggests the need for better coordination and more synergy between various sectors and stakeholders in the implementation of the regional strategy. The CCAs provided platforms for engaging and rallying national health stakeholders for action to strengthen health DRM and to create awareness about health DRM.

The VRAM and hospital safety assessment in Tanzania provided information which may be useful in improving health DRM and hospital safety. First, our evaluation showed that the most important disaster threats to the safety of health facilities were their functional capacity for effective coordination, contingency and business continuity planning, and the availability of medicines, supplies, and equipment for use during emergencies. Second, the results of the hospital safety evaluation provided information that may be useful in strengthening health facility disaster preparedness and risk reduction, as well as in improving the general day-to-day management of health facilities. Third, the VRAM exercise contributed to the identification of health sector disaster risks by hazards. The results showed that the most important causes of health vulnerability were factors external to the health sector, such as pre-disaster socio-economic status of affected people; therefore, a multi-sectoral approach to health systems and community resilience building is critically important.

### Study limitations

This study was subject to a number of limitations. First, the authors participated in the development, implementation, monitoring, and supervision of the regional strategy in various capacities and at different times over several years. Only a few authors were part of the entire process. Therefore, the selective, non-comprehensive memory of some of the authors might have biased the study findings and conclusions. The second limitation was the low response rate to the quantitative survey, which might not reflect the region’s true situation. Although no systematic difference was observed between countries that responded and those that did not, countries lagging behind in the implementation of the DRM strategies might have been more likely to have been among the non-responding countries.

Third, the self-reported and unsupervised nature of data collection for the quantitative survey might have introduced self-selective response biases with a tendency to overestimate the achievement or overstate the status of the relevant country’s progress. Fourth, the validity and reliability of the quantitative data collection tool has not been assessed; therefore, the questions might have been measuring different entities in different countries depending on subjective understanding or interpretation of the questionnaire.

These limitations raised potential data quality and validity issues. However, these were mitigated through discussions and validation of the findings with independent experts from the MS and the WHO. Furthermore, the findings of various HFA reports and DRM conference proceedings were used to validate some of the findings and conclusions of the study.

## Conclusions

The African regional strategy for health DRM has operationalized the HFA within the health sectors of participating African countries, and will provide impetus for implementation of the SFDRR in these health sectors in the post-HFA era. Although the implementation of the strategy was behind the anticipated targets, there were some positive outcomes. These included improved awareness and better understanding of health DRM concepts among health stakeholders across the region, and recognition of the need for DRR in the health sector. Significant effort is still required to ensure the gains made by the introduction of the strategy are nurtured to fruition. The ultimate achievement of the strategy’s goals will depend on its adaptability to evolving emergency scenarios in the post-HFA era. The lessons learned from its implementation to date may provide stimulus and opportunities to shape the post-HFA emergency public health agenda in the African region. Therefore, the strategy needs to be continually reviewed and aligned with the SFDRR to ensure that it remains relevant.

### Recommendations

Based on the findings of this study, we have proposed recommendations intended to facilitate accelerated implementation of the strategy, and constitute the main thrust of the post-2015 public health DRM agenda in Africa.

The principles of the Rio Declaration on Environment and Development state that *“human beings are at the centre of concerns for sustainable development; they are entitled to a healthy and productive life in harmony with nature”* [22]. The need to use people-centered approaches focused on harnessing human capital at all levels to build the resilience of disaster-prone and affected communities remains a central issue in the implementation of the regional strategy. Therefore, more investment will be required to build stronger DRM capacities within formal health sectors and also at the community level to foster community engagement and participation in health DRM programs. Appropriate strategies for effective community risk communication and strengthening community-based health initiatives are also required.

Lessons learned from the implementation of the regional strategy and available literature [23] support a health system-based approach to health DRM. Health DRM interventions should be integrated into longer-term health system strengthening programs at local and national levels [24]. This paradigm shift would reduce duplication, ensure sustainability, and increase funding opportunities for health DRM. The significance of timely, predictable, and sustainable funding for DRM (especially DRR) in the health sector is critical. Use of innovative approaches, such as expressly including DRR in the rationale for the existence of APHEF, or establishing a distinct funding mechanism for DRR/DRM is recommended. Broadening the scope of funding partners (private, public, local, international, multinational) that can contribute to the APHEF may be useful in ensuring that the yearly targets of the fund are met.

Multi-sectoral and multidisciplinary approaches should be used to mainstream health DRM into the DRM work of other sectors and vice versa. In this regard, future health sector DRM programs should be linked to, and synchronized with those of other relevant sectors such as water, sanitation, housing, and education, to ensure they address the social determinants of health that the CCAs have shown to contribute significantly to health vulnerabilities. Finally, available scientific evidence, health disaster risk information, and technological innovations should be compiled and used to guide the implementation of health DRM, especially in the areas of early warning, disaster response, and development of safer, smarter, greener, and resilient health facilities.

## Abbreviations

APHEF, African Public Health Emergency Fund; CCA, Country Capacity Assessment; DRM, disaster risk management; DRR, disaster risk reduction; EVD, Ebola Virus Disease; HeRAMS, Health Resources Availability Mapping System; HFA, hyogo framework for action; MOH, Ministry of Health; MS, member state; ODM, outbreak and disaster management; SFDRR, Sendai Framework for Action; VRAM, vulnerability and risk assessment and mapping; WHO, World Health Organization; WHO/AFRO, African Regional Office of the WHO

## Footnotes

1 Algeria, Angola, Benin, Botswana, Burkina Faso, Burundi, Cabo Verde, Cameroon, Central Africa Republic, Chad, Comoros, Congo, Cote d’Ivoire, Democratic Republic of the Congo, Equatorial Guinea, Eritrea, Ethiopia, Gabon, Gambia, Ghana, Guinea, Guinea Bissau, Kenya, Lesotho, Liberia, Madagascar, Malawi, Mali, Mauritania, Mauritius, Mozambique, Namibia, Niger, Nigeria, Rwanda, Sao Tome and Principe, Senegal, Seychelles, Sierra Leone, South Africa, South Sudan, Swaziland, Togo, Uganda, United Republic of Tanzania, Zambia, Zimbabwe
